# Arthroscopic coronal plane syndesmotic instability has been over-diagnosed

**DOI:** 10.1007/s00167-020-06067-5

**Published:** 2020-05-25

**Authors:** Noortje C. Hagemeijer, Mohamed Abdelaziz Elghazy, Gregory Waryasz, Daniel Guss, Christopher W. DiGiovanni, Gino M. M. J. Kerkhoffs

**Affiliations:** 1 Foot and Ankle Research and Innovation Lab, Department of Orthopaedic Surgery, Massachusetts General Hospital, Harvard Medical School, Boston, USA; 2 Department of Orthopaedic Surgery, Amsterdam University Medical Center, University of Amsterdam, Amsterdam Movement Sciences, Amsterdam Zuidoost, The Netherlands; 3 Academic Center for Evidence Based Sports Medicine (ACES), Amsterdam, The Netherlands; 4 Amsterdam Collaboration for Health and Safety in Sports (ACHSS), AMC/VUMC IOC Research Center, Amsterdam, The Netherlands; 5 Mansoura Faculty of Medicine, Mansoura University Hospital, Mansoura, Egypt; 6 Foot and Ankle Service, Department of Orthopaedic Surgery, Massachusetts General Hospital, Boston, USA; 7 Massachusetts General Hospital, Newton-Wellesley Hospital, Harvard Medical School, Boston, USA

**Keywords:** Ankle arthroscopy, Syndesmosis, Tibiofibular joint, Instability, Cut off

## Abstract

**Purpose:**

Ankle arthroscopy is widely used for diagnosis of syndesmotic instability, especially in subtle cases. To date, no published article has systematically reviewed the literature in aggregate to understand which instability values should be used intraoperatively. The primary aim was to systematically review the amount of tibiofibular displacement that correlates with syndesmotic instability after a high ankle sprain. A secondary aim is to assess the quality of such research.

**Methods:**

Systematic searches of EMBASE (Ovid) and MEDLINE via PubMed, CINAHL, Web of Science, and Google Scholar were used. Inclusion criteria: studies that arthroscopically evaluated the fibular displacement at various stages of syndesmotic ligament injury. Two reviewers independently extracted data and assessed methodological quality using the Anatomical Quality Assessment (AQUA) Tool and methodological index for non-randomized studies (MINORS).

**Results:**

Eight cadaveric studies and three clinical studies were included for review. All studies reported displacement in the coronal plane, four studies reported in the sagittal plane, and one reported findings in the rotational plane. Four cadaveric studies had a similar experimental set up and the weighted mean associated with instability in the coronal plane could be calculated and was 2.9 mm at the anterior portion of the distal tibiofibular joint and 3.4 mm at the posterior portion. Syndesmotic instability in the sagittal plane is less extensively studied, however available data from a cadaveric study suggests thresholds of 2.2 mm of posterior fibular translation when performing an anterior to posterior hook test and 2.6 mm of anterior fibular translation when performing a posterior to anterior hook test.

**Conclusions:**

The results have concluded that the commonly used 2.0 mm threshold value of distal tibiofibular diastasis may lead to overtreatment of syndesmotic instability, and that using threshold values of 2.9 mm measured at the anterior portion of the incisura and 3.4 mm at the posterior portion may represent better cut off values. Given the ready availability of 3 mm probes among standard arthroscopic instrumentation, at the very least surgeons should use 3 mm in lieu of 2 mm probes intraoperatively.

**Level of evidence:**

IV.

## Introduction

Isolated syndesmotic injuries occur in approximately 18% of all ankle sprains and 10–23% of all ankle fractures [4, 19, 25, 35, 54] and correlate with significantly poorer functional outcomes when left untreated [14, 42, 44]. The ankle draws much of its stability from its mortise structure, and instability of the distal tibiofibular ligamentous complex by definition allows this mortise to widen around the talus. The potentially altered tibiotalar relationship, in turn, can increase joint contact pressures potentiating post-traumatic arthritis [31, 41, 53]. Appropriate diagnosis and surgical repair of syndesmotic instability is, therefore, crucial towards preserving ankle stability and maximizing long term functional outcomes [23, 29].

MRI reliably detects syndesmotic injury, but as a static, unstressed modality, it is unable to reliably distinguish between stable and unstable injuries [22]. In contrast, ankle arthroscopy allows direct visualization of the distal tibiofibular articulation, both statically and under an applied stress load [38, 48]. While recent clinical and cadaveric studies have highlighted the role of ankle arthroscopy in diagnosing syndesmotic instability, the amount of fibular motion correlated with instability remains unclear as reported cut off values vary among. Most studies have highlighted a cut off value between 2 and 3 mm, but no published article has systematically reviewed these studies in aggregate to understand which values to use intraoperatively [7, 10, 30].

The primary aim of this study is to systematically review the published literature exploring the amount of fibular displacement found that correlates with syndesmotic instability after a high ankle sprain. A secondary aim is to assess the quality of such research. The clinical relevance of the present study is that it will provide an instability cut off value based upon a meticulous summary of all the available primary research for diagnosing syndesmotic instability arthroscopically which will be directly usable in the clinic and improve clinical outcome.

## Materials and methods

### Search strategy

Studies from the earliest recorded citations until June 18, 2019 were retrieved from the following electronic databases: EMBASE (Ovid) and MEDLINE via PubMed, CINAHL, Web of Science, and Google Scholar (Table 1). When searching through Google Scholar, only the first 250 results were exported because their search algorithm demonstrated that, despite thousands of results, the relevancy of these results quickly dropped. The search was conducted under the guidance of a clinical librarian.

**Table 1:** Search strategy and hits per electronic database

Database	Line		Items found	Unique hits
Pubmed	#1	(Arthroscop*[tiab] OR Arthroscopy[mesh]) AND (Syndesmos*[Title/Abstract] OR syndesmotic[Title/Abstract] OR tibiofibular*[Title/Abstract] OR “tibio fibular”[Title/Abstract] OR “high ankle”[Title/Abstract] OR AITFL[Title/Abstract] OR PITFL[Title/Abstract]) AND (“Wounds and injuries”[Mesh:noexp] OR “Sprains and strains”[Mesh] OR Rupture[Mesh:noexp] OR “Joint instability”[Mesh] OR “ankle injuries”[Mesh:noexp] OR Injur*[Title/Abstract] OR sprain*[Title/Abstract] OR instabilit*[Title/Abstract] OR unstable[Title/Abstract] OR rupture*[Title/Abstract] OR disruption*[Title/Abstract] OR tear*[Title/Abstract] OR torn[Title/Abstract])	126	125
Embase	#1	‘arthroscopy’/de OR ‘ankle arthroscopy’/de	159	54
	#2	arthroscop*:ab,ti		
	#3	#1 OR #2		
	#4	‘injury’/de OR ‘rupture’/de OR ‘ligament rupture’/de OR ‘joint instability’/de OR ‘sprain’/exp OR ‘ligament injury’/de OR ‘ankle injury’/de OR ‘syndesmotic injury’/de		
	#5	injur*:ab,ti OR sprain*:ab,ti OR instabilit*:ab,ti OR unstable:ab,ti OR rupture*:ab,ti OR disruption*:ab,ti OR tear*:ab,ti OR torn:ab,ti		
	#6	#4 OR #5		
	#7	‘syndesmosis’/exp		
	#8	syndesmos*:ab,ti OR syndesmotic:ab,ti OR tibiofibular*:ab,ti OR ‘tibio fibular’:ab,ti OR ‘high ankle’:ab,ti OR aitfl:ab,ti OR pitfl:ab,ti		
	#9	#7 OR #8		
	#10	#3 AND #6 AND #9		
CINHAL	S1	(MH “Arthroscopy”)	74	5
	S2	TI Arthroscop* OR AB Arthroscop*		
	S3	S1 OR S2		
	S4	TI (Syndesmos* OR syndesmotic OR tibiofibular* OR “tibio fibular” OR “high ankle” OR AITFL OR PITFL) OR AB (Syndesmos* OR syndesmotic OR tibiofibular* OR “tibio fibular” OR “high ankle” OR AITFL OR PITFL)		
	S5	(MH “Ankle Injuries”) OR (MH “Ankle Sprain+”) OR (MH “Sprains and Strains”) OR (MH “Wounds and Injuries”) OR (MH “Rupture”) OR (MH “Joint Instability”)		
	S6	TI (Injur* OR sprain* OR instabilit* OR unstable OR rupture* OR disruption* OR tear* OR torn) OR AB (Injur* OR sprain* OR instabilit* OR unstable OR rupture* OR disruption* OR tear* OR torn)		
	S7	S5 OR S6		
	S8	S3 AND S4 AND S7		
Web of science	#1	TOPIC: (Arthroscop*)	144	52
	#2	TOPIC: (Syndesmos* OR syndesmotic OR tibiofibular* OR “tibio fibular” OR “high ankle” OR AITFL OR PITFL)		
	#3	TOPIC: (Injur* OR sprain* OR instabilit* OR unstable OR rupture* OR disruption* OR tear* OR torn)		
	#4	#1 AND #2 AND #3 Indexes=SCI-EXPANDED, SSCI, A&HCI, CPCI-S, CPCI-SSH, BKCI-S, BKCI-SSH, ESCI, CCR-EXPANDED, IC Timespan=All years		
Google scholar	#1	Arthroscopy | arthroscope | Syndesmoses | syndesmosis | syndesmotic | tibiofibular | “tibio fibular” | “high ankle” | AITFL | PITFL Injury | injuries | sprain | sprains | instability | unstable | rupture | disruption | tear | torn	500	252
Total			1003	488

### Eligibility criteria

All the studies that arthroscopically evaluated fibular displacement in the three planes after different type of ligamentous injuries were considered for inclusion. All randomized controlled trials, controlled non-randomized trials, prospective and retrospective cohort studies and case series were included. Animal studies and review studies were excluded, though the references of related review articles were assessed for any additional eligible studies. No age restrictions were applied.

### Variables and target outcome

The target variables included, (1) the threshold considered to represent an unstable syndesmosis, (2) fibular displacement in the coronal, sagittal, and rotational plane in mm or degrees, (3) associated injuries, (4) location of the measurement, and (5) type of stress test. Associated injuries were defined as injuries to the ligamentous structures of the syndesmosis the anterior inferior tibiofibular ligament (AITFL), the interosseous ligament (IOL), and the posterior inferior tibiofibular ligament (PITFL), the lateral fibulo-talo-calcaneal ligament complex LFTCL, consisting of the anterior tibiofibular ligament (ATFL), calcaneofibular ligament (CFL), posterior talofibular ligament (PTFL) [51], the deltoid ligament (DL), and concomitant ankle fractures. Other reported diagnostic tools (radiographs, CT, MRI, or ultrasound) and intra- and inter-rater reliability scores were also recorded.

### Reference standard

In cadaveric studies the ligamentous injury pattern was used as a reference when comparing the amount of fibular displacement across studies. Syndesmotic instability was defined as an injury that was associated with tibiofibular displacement significantly different from the intact state. In In vivo studies, this comparison cannot be made and, therefore, the threshold considered to represent an unstable syndesmosis and associated injuries was described descriptively for each study.

### Study selection

Two authors (NH and MA) independently screened titles and abstracts, using predetermined inclusion and exclusion criteria with the help of Covidence, https://www.covidence.org/home. Disagreement was resolved by an attempt to reach consensus. In cases where no consensus was reached, a third reviewer (GW) was consulted to resolve the disagreement.

### Data extraction

Data were extracted by one reviewer (NH) and thereafter checked by all co-authors. Extracted data were collected in a predefined format from Microsoft Excel for Mac (version 15.37). Study design, patient or cadaver characteristics, arthroscopic measurement details, diastasis measurements or pre-determined cut off values, related injuries, and other radiographic outcomes were extracted.

### Quality assessment

Methodological quality of the cadaveric studies was assessed using the Anatomical Quality Assessment (AQUA) Tool by two independent reviewers (NC and MA) [18]. This tool is designed to help evaluate the performed experiment, i.e. the arthroscopic diagnosis of syndesmotic instability, by addressing five key domains: (1) whether objectives were clearly defined and appropriate, (2) whether the study design was appropriate for answering the aims, (3) whether the methodology was described in sufficient detail to permit replication, (4) whether the anatomical definitions were accurately defined and described, and (5) whether the results were accurately calculated and reported. The methodological quality of included studies was assessed using the Methodological Index for Non-Randomized Studies (MINORS) instrument [46]. MINORS is an instrument designed to assess methodological quality of both non-comparative and comparative studies. For this study, only the non-comparative factors of the MINORS instrument were used. Disagreement was resolved by consensus or third-party adjudication (GW).

### Statistical analysis

For each study, the reported amount of distal tibiofibular displacement, associated injury patterns, and related pathologies were recorded and summarized. Statistical heterogeneity was then determined using the Higgins and Thompson *I*^2^ index as well as a chi-squared test to assess for heterogeneity. The *I*^2^ was considered to be of low heterogeneity when < 0.25, moderate heterogeneity when between 0.25 and 0.50, and high heterogeneity when > 0.50. A fixed model was used when heterogeneity was low or moderate. If the data heterogeneity was high, a formal meta-analysis would not be performed and instead results would be presented in a descriptive manner along with weighted means and SDs when able. In case of unavailable raw data the range of the means would be provided instead. *P*-values of < 0.05 were considered significant. All analyses were performed with Stata 13.0 for Mac (StataCorp LP, College Station, TX, USA).

## Results

**Fig. 1:**
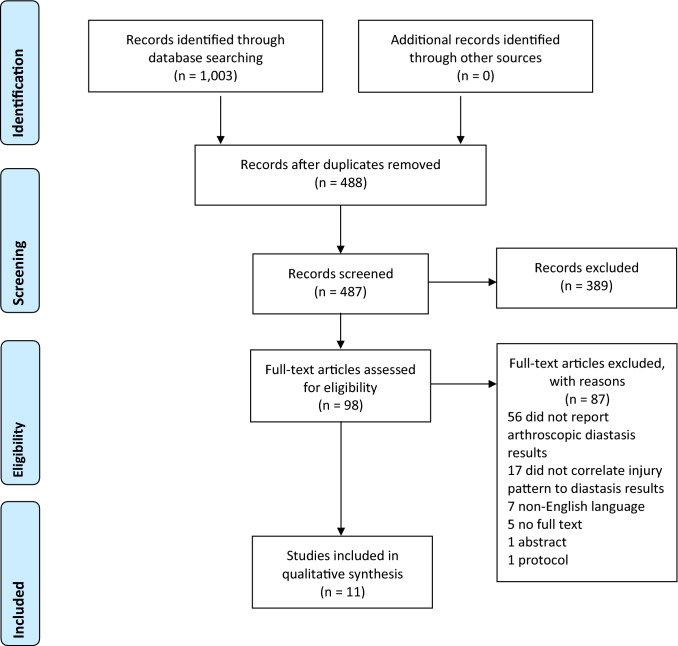
PRISMA (preferred reporting items for systematic meta-analyses) flowchart for study inclusion. *RCT* randomized controlled trial

A total of 1003 studies were identified (Fig. 1). Of these studies, 515 were duplicates and removed prior to the first round of selection. Of those remaining, 389 articles were excluded based upon title and abstract screen and 98 articles were selected for full-text screening. A total of 8 cadaveric studies and three clinical studies were included in this systematic review [12, 15, 16, 26, 27, 30, 31, 50, 53, 56]. Three studies’ corresponding authors were contacted by email for additional information but were not ultimately included due to non-response [40, 49, 52].

### Variables and target outcome

The threshold that was considered to represent an unstable syndesmosis, amount of displacement, associated injury patterns, and arthroscopy technique details per each study are summarized in Table 2.

**Table 2:**
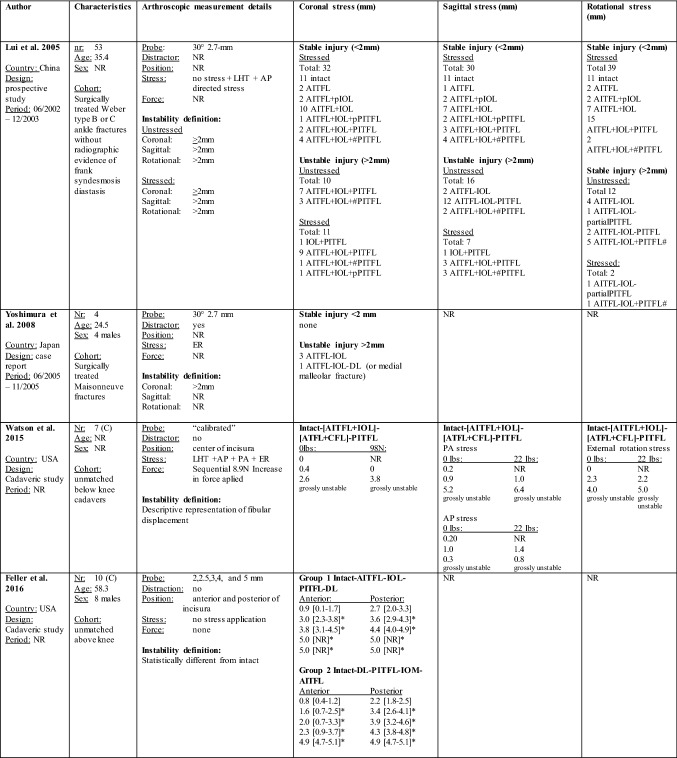

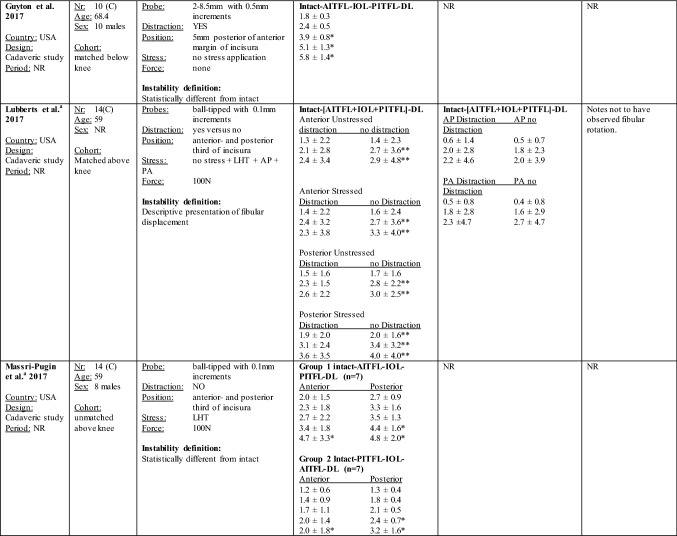

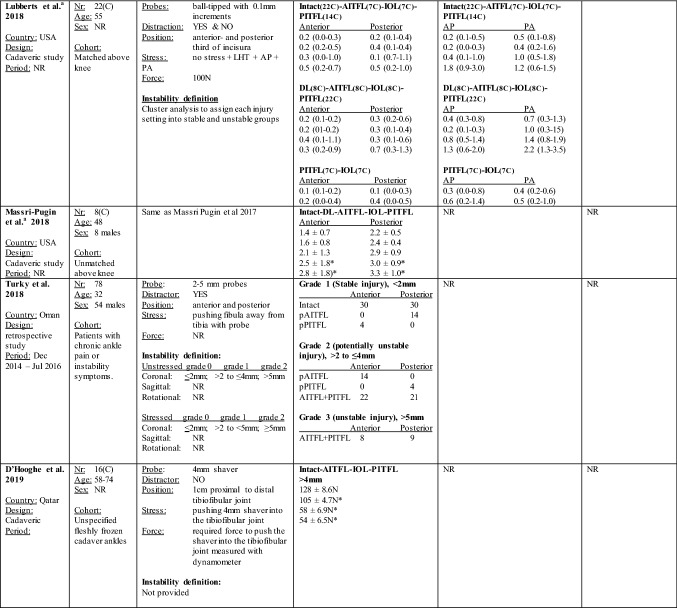
Arthroscopic syndesmotic instability measurements per study

Syndesmotic instability measurements obtained with the arthroscope expressed in mean ± SD, mean [95% CI], or median(IQR) in millimeters per ligament transection stage, stress condition, probe position, and with- or without traction application. Values listed which derived from cadaveric studies correlate with the injury order as stated from top to bottom

*mm* millimeters, *nr* number, *NR* not reported, *LHT* lateral hook test, *AP* anterior to posterior, *PA* posterior to anterior, *AITFL* anterior inferior tibiofibular ligament, *IOL* interosseous ligament, *PITFL* posterior inferior tibiofibular ligament, *DL* deltoid ligament, *ATFL* anterior talofibular ligament, *CFL* calcaneal talofibular ligament, *p* partial, *#* fracture, *USA* United States of America, *C* Cadaver, *N* Newton, *lbs* pounds

*Significant difference from intact

**Significant difference as compared to distraction

^a^Studies derived from the same experimental set up

All cadaveric studies reported on fibular displacement in the coronal plane. Significant tibiofibular displacement from the intact state ranged from 1.6 to 4.4 mm at the anterior and from 2.4 to 4.4 mm at the posterior third of the incisura [15, 27, 31, 32, 53]. One study provided an instability threshold based upon a cluster analysis, which was 2.6 mm when measuring at the anterior third coronal plane space of the incisura, and 3.4 mm when measuring at the posterior third space [26]. The other two cadaveric studies did not compare tibiofibular displacement to the reference intact state [12, 27]. Due to a high heterogeneity, a formal meta-analysis was not performed and weighted means for syndesmotic instability in the coronal plane were calculated instead for those cadaveric studies who had a similar experimental set up including probe positioning, method of stress application, and the absence of use of an ankle distractor [15, 27, 31, 32]. The weighted mean of syndesmotic instability in the coronal plane with a lateral fibular stress maneuver was 2.9 mm at the anterior portion of the incisura and 3.4 mm at the posterior portion of the incisura. Weighted means of syndesmotic instability and per each injury pattern are provided in Table 3.

**Table 3:** Weighted means of the arthroscopic diastasis measurements in the coronal plane

Number of transected ligaments	Anterior incisura (range of means) (mm)	Posterior incisura (range of means) (mm)
Intact	1.4 (0.8–2)	2.1 (1.3–3.3)
One ligament	2.0 (1.4–3)	2.6 (1.6–3.6)
Two ligaments	2.4 (1.7–3.8)	3.3 (2.1–4.4)
Three ligaments	3.0 (2–5)	3.7 (2.4–5)
Syndesmotic instability value*	2.9 (1.6–4.7)	3.4 (2.4–4.4)

*The syndesmotic instability weighted mean value was calculated using the tibiofibular displacement values from those injury patterns that showed a significant difference from the intact state

All three in vivo studies reported on displacement in the coronal plane. Two of these chose to use the threshold of > 2 mm for diagnosing and treating syndesmotic instability [30, 56]. The other study categorized each injury pattern based upon the diastasis in a self-made grading scheme where they considered < 2 mm stable, > 2 to < 5 mm potentially unstable, and > 5 mm unstable [50].

Three cadaveric studies reported on tibiofibular displacement values in the sagittal plane [26, 27, 53]. One study provided an instability threshold based upon a cluster analysis which was 2.2 mm when pulling anteriorly, and 2.6 mm when pulling posteriorly [26]. Two studies presented their results descriptively [27, 53].

Only one In vivo study reported on displacement in the sagittal plane, handling a threshold of > 2 mm for diagnosing and treating syndesmosis instability [30].

None of the cadaveric studies reported findings in the rotational plane. One clinical study reported findings in the rotational plane for which they handled a threshold of > 2 mm [30].

Feller et al. and Lui et al. reported concomitant radiographic measurements for each injury pattern, which are presented in Table 4 [15, 30].

**Table 4:** Fluoroscopic syndesmotic instability measurements per study

Author	Cohort and methods	Measurements		
		TFO (mm)	TFCS (mm)	MCS (mm)
Lui et al. (2005) Country: China Design: prospective study Period: 06/2002–12/2003	Nr: 53 Age: 35.4 Sex: NR Cohort: surgically treated Weber type B or C ankle fractures without radiographic evidence of frank syndesmosis diastasis	NR	Injury patterns which did not show widening: 11 Intact 2 AITFL 2 AITFL + IOL_partial_ 9 AITFL + IOL 1 IOL + PITFL 2 AITFL + IOL + _p_PITFL 4 AITFL + IOL + PITFL 5 AITFL + IOL + PITFL# Injury patterns which did show widening: 14 AITFL + IOL + PITFL 3 AITFL + IOL + PITFL#	NR
Feller et al. (2016) Country: USA Design: Cadaveric study Period: NR	Nr: 10 (C) Age: 58.3 Sex: 8 males Cohort: unmatched above knee	Group 1 Intact-AITFL-IOL-PITFL-DL 4.9 (4.3–5.6) 4.4 (3.63–5.7) 2.8 (2.4–3.2)* 1.5 (0.1–2.9)* < 0 (NR)	Group 1 Intact-AITFL-IOL-PITFL-DL 4.7 (3.5–5.9) 5.4 (4.5–6.3)* 5.6 (4.8–6.4) 7.53 (5.9–9.2) 11.46 (10.1–12.8)*	Group 1 Intact-AITFL-IOL-PITFL-DL 4.0 (3.1–4.9) 4.0 (3.3–4.6) 5.0 (3.9–6.2) 5.7 (4.5–7.0)* 9.8 (8.11–11.5)*
		Group 2 Intact-DL-PITFL-IOM-AITFL 4.7 (3.5–5.9) 3.8 (3.0–4.6) 3.0 (2.0–4.0)* 2.2 (1.6–2.7)* <0 (NR)	Group 2 Intact-AITFL-IOL-PITFL-DL 5.5 (5.0–6.0) 5.4 (4.3–6.5)* 5.3 (4.3–6.4) 6.1 (4.8–7.3) 11.1 (9.3–12.9)*	Group 2 Intact-AITFL-IOL-PITFL-DL 4.8 (3.9–5.7) 4.9 (4.2–5.7) 5.64 (5.0–6.3) 5.8, (4.7–6.8)* 10.3, (4.7–6.8)*

*TFO* tibiofibular overlap, *TFCS* tibiofibular clear space, *MCS* medial clear space, *mm* millimeters, *nr* number, *NR* not reported, *AITFL* anterior inferior tibiofibular ligament, *IOL* interosseous ligament, *PITFL* posterior inferior tibiofibular ligament, *DL* deltoid ligament, *p* partial, # fracture, *C* Cadaver

*Significant difference from intact

Two clinical studies reported on in vivo cartilage damage in the setting of syndesmotic instability [50, 56]. Turky et al. [50] reported that over 90% of the patients had additional lesions also including ATFL injuries. All four patients described by Yoshimura et al. [56] had talar lesions on the posteromedial aspect of the talar dome.

There were two cadaveric studies which included an inter-observer agreement analysis as part of their study methodology, which both derived from the same experimental set up, by having two observers assess three specimens independently [26, 27]. Substantial agreement was found for anterior third coronal plane tibiofibular diastasis and sagittal plane tibiofibular translation. Moderate agreement was found for posterior third coronal plane tibiofibular diastasis.

### Quality assessment

All but two studies had a high risk of bias due to a methodology that was not described in sufficient detail to permit replication as per the AQUA tool described above (Table 5). This specifically pertained to a failure to undertake appropriate measures to reduce inter- and intra- observer variability. The only studies included in this review that explicitly analyzed the reliability of the measurements were the studies of Lubberts et al. [26, 27]. The methodological quality of the clinical studies was graded according to the MINORS criteria (Table 6) and the average score was 6.7 out 16 points (41.7% of maximum).

**Table 5:**
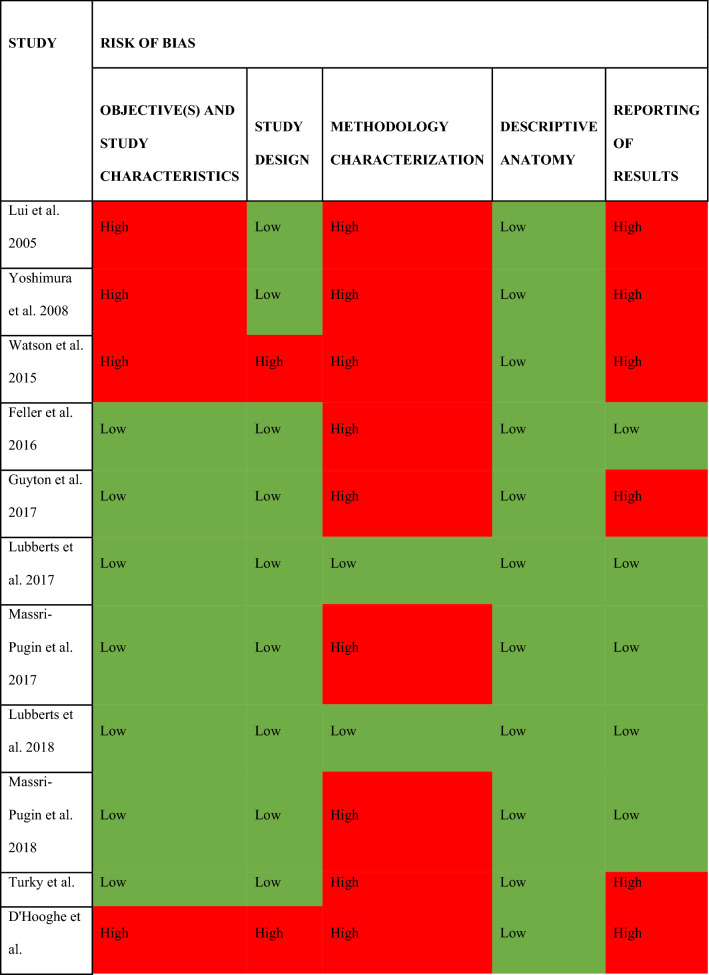
Summary table for the risk of bias across the included studies

High risk: red, low risk: green, unclear risk: blue

**Table 6:** Quality assessment of the included clinical studies using the MINORS criteria

Authors	Year	Journal	Evidence	Study design	1	2	3	4	5	6	7	8	Total
Lui et al.	2005	Arthroscopy	II	Cohort study	0	2	1	1	0	0	0	0	6
Yoshimura et al.	2008	Orthopaedic science	IV	Case series	2	1	0	2	0	1	0	0	7
Turky et al.	2018	Foot and ankle surgery	III	Cohort study	2	2	2	1	0	0	0	0	7

Only the non-comparative part of the MINORS criteria was used (i.e. first 8 questions). The criteria of Methodological Index for Non-Randomized Studies (MINORS) with 0 points when not reported, 1 when reported but not adequate, and 2 when reported and adequate. Maximum score is 16

1. A clearly stated aim: the question addressed should be precise and relevant in the light of available literature

2. Inclusion of consecutive patients: all patients potentially fit for inclusion (satisfying the criteria for inclusion) have been included in the study during the study period (no exclusion or details about the reasons for exclusion)

3. Prospective collection of data: data were collected according to a protocol established before the beginning of the study

4. End points appropriate to the aim of the study: unambiguous explanation of the criteria used to evaluate the main outcome which should be in accordance with the question addressed by the study. In addition, the end points should be assessed on an intention-to-treat basis

5. Unbiased assessment of the study end point: blind evaluation of objective end points and double-blind evaluation of subjective end points. Otherwise, the reasons for not blinding should be stated

6. Follow-up period appropriate to the aim of the study: the follow-up should be sufficiently long to allow the assessment of the main endpoint and possible adverse events

7. Loss to follow-up less than 5%: all patients should be included in the follow-up. Otherwise, the proportion lost to follow-up should not exceed the proportion experiencing the major end point

8. Prospective calculation of the study size: information of the size of detectable difference of interest with a calculation of 95% CI, according to the expected incidence of the outcome event, and information about the level for statistical significance

## Discussion

The most important finding of this study is that the commonly used threshold of 2.0 mm potentially leads to overtreatment and using 3.0 mm of tibiofibular diastasis measured at the anterior portion of the incisura or 3.4 mm of tibiofibular diastasis at the posterior portion seems to be a better cut off value.

Syndesmotic instability can cause significant long-term morbidity if undiagnosed and even subtle persistent syndesmotic instability can already be very disabling. The latter can be difficult to appreciate with clinical maneuvers or with static imaging. This has generated increasing interest in directly visualizing the distal tibiofibular articulation arthroscopically. Despite the fact that ankle arthroscopy has been proposed as the gold standard for diagnosing (subtle) syndesmotic instability, no prior literature review has systematically evaluated the available published research detailing arthroscopic examination of syndesmotic instability [16]. In total, 11 studies were ultimately included in this review, though high heterogeneity did not allow a formal meta-analysis.

Syndesmotic instability is inherently multidimensional and is comprised of tibiofibular diastasis in the coronal plane, fibular translation in the sagittal plane, and fibular external rotation [5, 20]. The majority of the published literature, however, evaluates the syndesmosis primarily in the coronal plane while applying a lateral fibular “hook test”. Several cut off values have been proposed by various authors, including 1 mm with stress application [11], > 2 mm without stress application [17], > 2 mm with stress application [7, 8, 21, 28, 49], > 3 mm without stress application [52], > 3 mm with stress application [1, 11, 38], > 4 mm without stress application [8], and > 4 mm with stress application [43]. Most studies used 2 mm as a cut off value, but this may over-diagnose syndesmotic instability and 3 mm may instead serve as a better cut off value in the coronal plane [3, 15, 16, 26, 31, 32, 53]. In this review, the weighted mean of syndesmotic instability in the coronal plane with a lateral fibular stress maneuver was 2.9 mm at the anterior portion of the incisura and 3.4 mm at the posterior portion of the incisura.

Syndesmotic instability in the sagittal plane is less well-described in the arthroscopic literature. Those that did investigate sagittal plane instability found that the instability is more visible in the sagittal plane than in the coronal plane in the setting of an unstable syndesmosis [6, 26, 27, 30, 38, 45, 53, 55]. However, the total amount of sagittal plane fibular translation that best serves as a clinical threshold for diagnosing syndesmotic instability remains uncertain. Lubberts et al. created a prediction model based on cluster analysis of data from a cadaveric syndesmotic injury model, which incorporated coronal as well as the sagittal plane measurements for assessing syndesmotic instability [26]. They reported cut off values of 2.2 mm of posterior fibular translation when performing an anterior to posterior hook test and 2.6 mm of anterior fibular translation when performing a posterior to anterior hook test [26].

Rotational plane stability is rarely assessed arthroscopically in the published literature. One clinical study included in this systematic review evaluated the rotation by assessing the difference between the distance from the anterior border to the incisura and the distance between the posterior border and the incisura [30]. Given that this value can be confounded by concomitant coronal and sagittal plane translation, the arthroscope may not be the preferred method for determining fibular rotation [30].

Technical factors also influence the amount of tibiofibular diastasis visualized arthroscopically, including (1) the amount of stress applied and in which direction [12, 26, 39, 47], (2) whether a distractor is being used [27], and (3) where in the incisura the diastasis is being measured [15, 26, 31, 32]. Stoffel et al. highlighted that stress forces above 100 N do not result in additional diastasis, and, therefore, numerous studies have standardized a 100 N force applied to the fibula 5 cm proximal to the tibiotalar joint in either the coronal or sagittal planes [26, 27, 31, 32, 47]. Furthermore, an ankle distractor is almost universally employed during arthroscopic procedures to the ankle, but this traction can mask syndesmotic instability, likely due to the applied tension to the surrounding intact ligaments and other soft tissues. Distraction should, therefore, be released at the time of measurement, especially if the syndesmotic instability is anticipated to be subtle [15, 27, 37]. Last, measurements in the posterior third of the incisura may result in higher values than those anteriorly [15, 31, 32].

It is important to note that the reported distinction between a stable and unstable syndesmotic measurement value in the literature, as assessed arthroscopically, is a statistical one. The threshold values for instability, as discussed above, are those in which an injury to the syndesmosis has allowed the fibula to translate, either coronally or sagittally, on average significantly more than in the intact state. On the other hand, the degree of diastasis or translation that has clinical implications remains unclear and may or may not entirely correlate with the discussed values. Determination of the clinical effect of the various cut off values will be challenging given that it would require a randomized controlled or a multi-center observational study in which different surgeons use different thresholds.

Ankle arthroscopy does also have some inherent disadvantages. It is an invasive technique and, consequently, available to a select group of patients with either a high level of pre-operative suspicion or patients with a concomitant fracture that independently require surgery. Furthermore, unlike imaging modalities, arthroscopy cannot benefit from using the contralateral side as an internal control, which becomes increasingly useful as instability becomes more subtle, especially in chronic injury scenarios [13, 24, 36]. Diagnostic techniques that are non-invasive, dynamic, and allow for a bilateral examination at the same time will therefore almost undoubtedly play an increasing role in diagnosing syndesmotic instability in the future alongside the arthroscope. Modalities such as weightbearing CT or dynamic ultrasound fit the above criteria, and their roles should be further explored in both biomechanical and clinical studies [2, 5, 33, 34].

Two papers included in this review also assessed radiographic or fluoroscopic measurements [15, 30]. They corroborated other radiographic studies highlighting that parameters such as tibiofibular overlap, tibiofibular clear space and medial clear space, do not seem sufficiently sensitive to diagnose syndesmotic instability [9, 22].

This review has some limitations. The overall quality of the included studies was low and there was a high risk of bias. For the experimental studies the low quality was most commonly due to a lack of intra- and inter-observer reliability measurements. Secondly, this study used the AQUA assessment tool, which is specifically designed for evaluating the methodology of an anatomic experiment and was therefore deemed most applicable for inclusion of cadaveric studies, but this tool is only now undergoing the process of being formally validated [18]. Last, ligamentous injury pattern was used as a reference standard when comparing the amount of fibular displacement across studies for the same injury and was used to calculate the weighted means. It should be noted that the injury pattern seen in a clinical setting does not invariably correlate with instability [30, 50, 56]. Clinical instability likely also relies on other potential patient factors (e.g. age, weight, and chronicity), but it may also result from a measurement bias given that the forces used in the various stress tests used are often not reported in the literature.

## Conclusion

The results have concluded that the commonly used 2.0 mm threshold value of distal tibiofibular diastasis may lead to overtreatment of syndesmotic instability, and that using threshold values of 2.9 mm measured at the anterior portion of the incisura and 3.4 mm at the posterior portion may represent better cut off values. Given the ready availability of 3 mm probes among standard arthroscopic instrumentation, at the very least surgeons should use 3 mm in lieu of 2 mm probes intraoperatively.

## Abbreviations

AITFL: Anterior interior tibiofibular ligament
AQUA: Anatomical quality assessment tool
ATFL: Anterior tibiofibular ligament
CFL: Calcaneofibular ligament
CINHAL: Cumulative index to nursing and allied health literature
DL: Deltoid ligament
IOL: Interosseous ligament
LFTCL: Lateral fibulo-talo-calcaneal ligament complex
PITFL: Posterior interior tibiofibular ligament
PTFL: Posterior talofibular ligament
